# The Anterior Intermeniscal Ligament: A Comparative Study of Morphology in Male and Female Cadavers

**DOI:** 10.7759/cureus.84021

**Published:** 2025-05-13

**Authors:** Nicholas A Tchekryguin, M. Cord Neal, Ethan Blythe, Blake Archer, Chakravarthy Sadacharan, Samantha P Tippen

**Affiliations:** 1 Medicine, Tilman J. Fertitta Family College of Medicine, Houston, USA; 2 Anatomy, Tilman J. Fertitta Family College of Medicine, Houston, USA; 3 Anatomy, Baylor College of Medicine, Houston, USA

**Keywords:** anatomy and physiology, anterior intermeniscal ligament, knee, knee biomechanics, orthopedic procedures

## Abstract

Introduction: The anterior intermeniscal ligament (AIML) is a highly prevalent and functionally debated anatomical structure within the knee joint. While previous studies have suggested its potential role in meniscal stability, load distribution, and proprioception, detailed anatomical analyses are limited. This study aimed to assess the architectural characteristics of the AIML in male and female cadavers to elucidate potential sex-based differences.

Methods: This observational study was based on 112 available cadaveric knee specimens, with a female-to-male ratio of 1:1, sourced from the anatomy labs of the Tilman J. Fertitta Family College of Medicine and Baylor College of Medicine. After blunt dissection, the AIML was exposed and measured using a Mitutoyo digital caliper 500-172-30 (Takatsu-ku, Kawasaki, Kanagawa, Japan). Various measurements of the AIML and surrounding soft tissue structures were performed, and the recorded averages were organized in Microsoft Excel (Microsoft Corporation, Redmond, Washington, United States). Statistical analysis was conducted utilizing GraphPad Prism 10.2.0 (GraphPad Software, Inc., San Diego, California, United States).

Results: Statistical analyses revealed almost no significant differences in AIML dimensions other than greater medial AIML thickness in the right knee of male cadavers compared to female cadavers (right knee: t(110)=2.14; p=0.04). In the left knee, male cadavers demonstrated a trend toward increased medial AIML thickness, though this did not reach statistical significance (left knee: t(110)=-1.18; p=0.24). Additionally, male cadavers exhibited a trend toward larger mid-point AIML thickness bilaterally compared to female cadavers (right knee: t(110)=1.65 and p=0.10; left knee: t(110)=1.48 and p=0.14). These findings may suggest that sex-related factors influence AIML morphology, though further research with a larger sample size is necessary to determine statistical significance.

Conclusions: The absence of significant anatomical differences between sexes illuminates a potential contribution to sex-based disparities in knee pathology. Understanding AIML's structural properties may have implications for meniscal and anterior cruciate ligament (ACL) surgeries, improving surgical precision and patient outcomes. Future studies could investigate other sex-related factors, such as q-angles or estrogen exposure, and their effect on AIML morphology. Additionally, other avenues of research could investigate and compare morphological measurements in younger and older populations to evaluate whether this structure is sensitive to osteoarthritic insults, better defining its functional role in knee joint stability.

## Introduction

The anterior intermeniscal ligament (AIML) is an anatomical structure located posterior to the patellar fat pad and directly inferior to the intercondylar notch of the knee. It connects the anterior horns of the lateral and medial menisci as a slender band of connective tissue. The AIML is highly prevalent, reported in 34-94% of individuals, with variable insertion patterns [1-5]. While its function remains debated, it is believed to help stabilize the menisci and limit anterior-posterior movement during varying degrees of knee flexion [6]. 

Research by Paci et al. and Ollivier et al. suggests that the AIML reduces axial contact pressures in the medial compartment of the knee during flexion [7,8]. However, these findings contrast with those of Poh et al., who found no statistically significant increase in tibiofemoral contact pressure following AIML removal, suggesting that the menisci may function independently of the AIML in transforming axial loads into circumferential hoop stresses [9]. 

Histological studies have revealed a rich network of free nerve endings, blood vessels, and Ruffini corpuscles within the AIML, indicating a potential role in knee proprioception [10,11]. This view is further supported by Guess et al., who highlighted AIML's dense neural innervation as evidence of its proprioceptive role while also demonstrating diminished meniscal load-sharing of knee joint forces after its removal [12]. 

Despite these insights, the full role of the AIML remains incompletely understood, though it potentially contributes to both biomechanical and sensorimotor functions within the knee. While much research has focused on defining its primary function, there is a lack of precise models and data illustrating AIML's spatial relationships with surrounding soft tissue structures. To address this gap, our study aims to compare the architectural characteristics of the AIML between men and women, providing insights that may enhance surgical success in meniscal and anterior cruciate ligament (ACL) procedures, ultimately improving patient outcomes.

## Materials and methods

Study design 

This descriptive anatomical study was conducted on 112 cadaveric knee specimens obtained from the anatomy laboratories of Tilman J. Fertitta Family College of Medicine and Baylor College of Medicine in Houston, Texas. The primary objective was to analyze the dimensions and anatomical variations of the AIML.

The specimens were preserved in phenol and stored under standard control conditions to maintain tissue integrity for dissection and measurement. The cadavers had an average age of 77 years (range: 45-95 years). The sample size was determined by practical availability, and a convenience sampling method was employed, including all cadaveric knee specimens that met the inclusion criteria at the time of data collection.

Inclusion and exclusion criteria 

Inclusion criteria required specimens to have an intact AIML with surrounding soft tissue structures, clearly identifiable menisci, and well-preserved tissues with minimal degradation. Specimens were excluded if they had been significantly altered by prior anatomical dissection or if they had undergone surgical interventions (e.g., total knee replacements) that distorted the region of interest.

To maintain specimen integrity, all dissections were performed following a standardized protocol, ensuring the maximal preservation of cadaveric structures. For previously dissected specimens, three independent researchers verified the integrity of the AIML and surrounding tissues before data collection.

Data collection 

Dissections were performed by trained researchers following a standardized protocol to minimize variability. Each researcher independently verified the presence of key anatomical structures before initiating measurements.

The dissection process began at the distal patella, focusing on the attachment to the tibial tuberosity to expose the AIML. The lateral and medial joint capsule structures were carefully transected to reveal the underlying patellar fat pad. Blunt dissection was then used to further expose the AIML, with any remaining adipose tissue carefully removed to ensure clear visualization.

Measurements were taken using a Mitutoyo digital caliper 500-172-30 (Takatsu-ku, Kawasaki, Kanagawa, Japan), with each measurement recorded twice and averaged to enhance accuracy. The length of the AIML was measured from the most medial point of the lateral anterior meniscal horn to the transition point of the medial anterior meniscal horn. At these transition points, the thickness of the respective meniscal horns was measured just lateral to the AIML.

Medial and lateral thickness measurements of the AIML were also recorded. The midpoint thickness of the AIML was measured at approximately half its length. From this midpoint, the anterior portion of the AIML was measured to the root of the ACL at the anteromedial aspect of the intercondylar area. The same measurement technique was applied to the posterior horn of the menisci relative to the AIML. To ensure measurement consistency, the knee was positioned at a 45-degree flexion angle throughout the procedure. This study was deemed IRB-exempt under federal regulations.

Quality assurance

A standardized dissection protocol was practiced on prior cadavers to refine dissection techniques and ensure the uniform exposure of the AIML before data collection. To enhance measurement reliability, two independent researchers measured the AIML dimensions and its distance to surrounding soft tissue structures.

Discrepancies exceeding 0.5 mm for thickness measurements and 2 mm for distance measurements were flagged for reevaluation. In cases where discrepancies persisted, a third researcher performed an independent measurement to reach a consensus. All measurements were recorded twice and averaged to minimize observational bias and ensure data accuracy.

Statistical analysis 

The recorded averages were organized in Microsoft Excel (Microsoft Corporation, Redmond, Washington, United States), and statistical analysis was conducted utilizing GraphPad Prism 10.2.0 (GraphPad Software, Inc., San Diego, California, United States). 

## Results

AIML length

**Figure 1:**
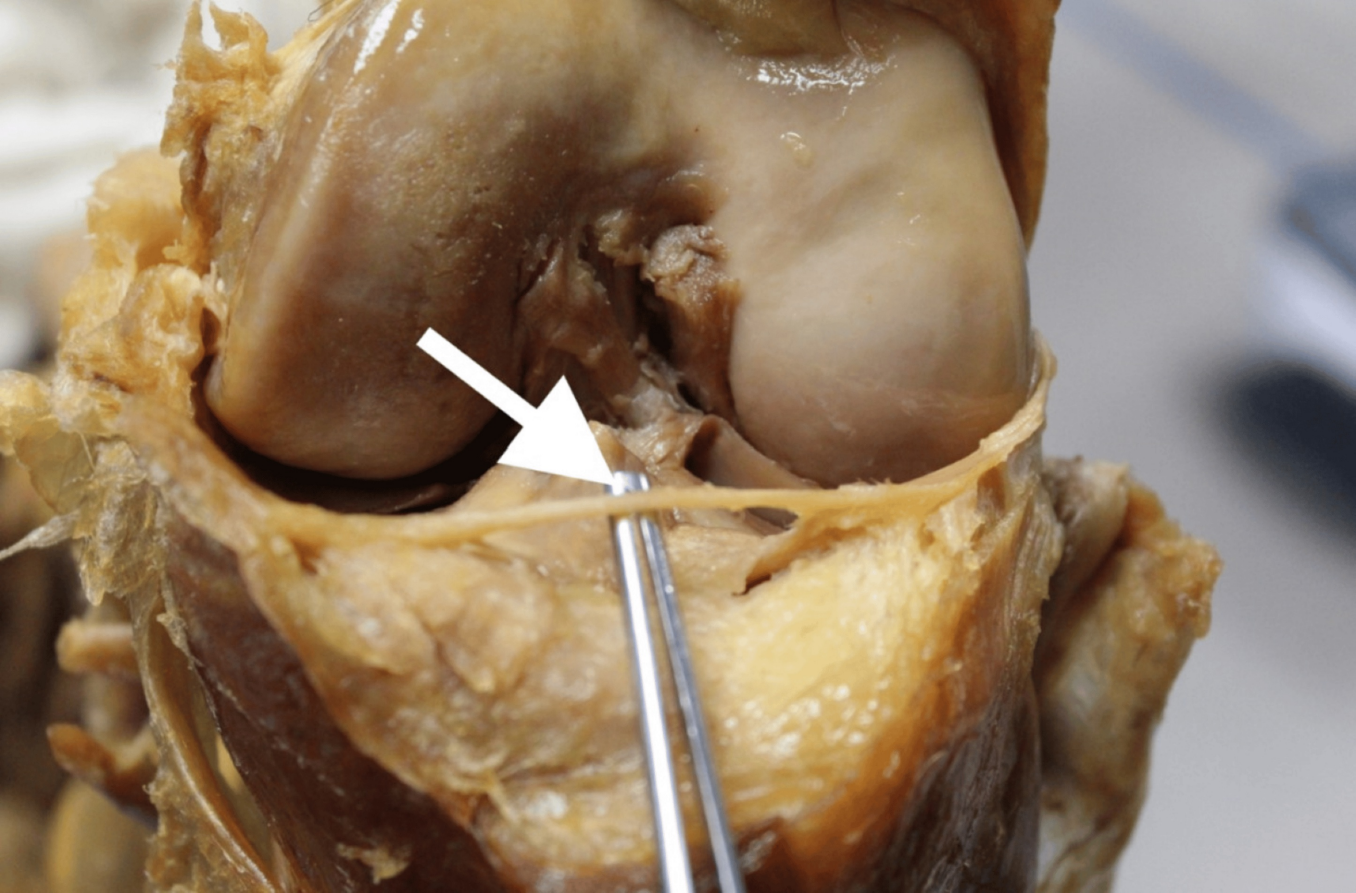
Anterior view of the right knee with the AIML exposed AIML: anterior intermeniscal ligament

**Figure 2:**
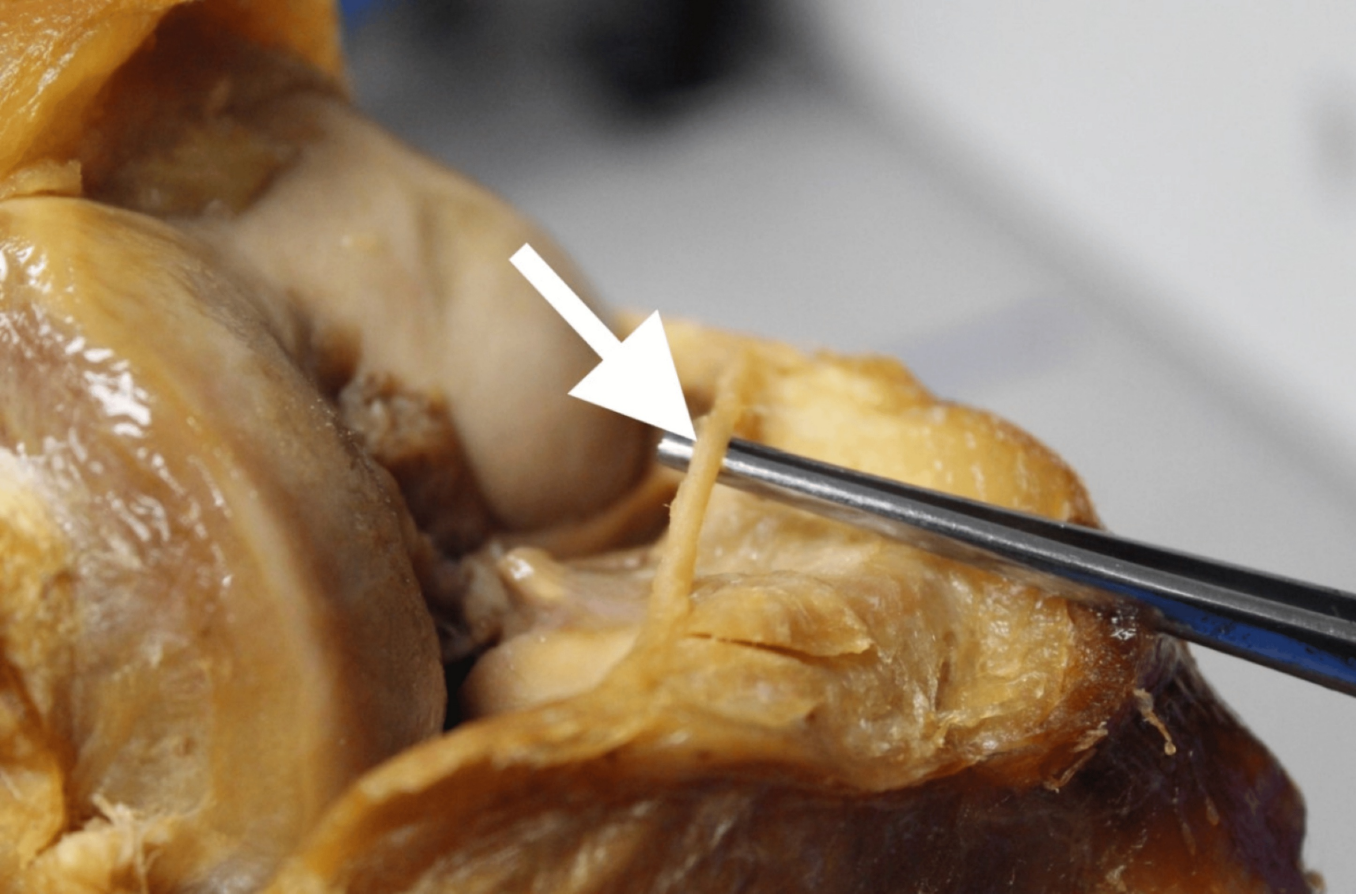
Lateral view of the right knee with the AIML exposed AIML: anterior intermeniscal ligament

Using unpaired t-tests, we found no statistically significant difference in AIML length between male and female knee specimens; however, certain trends were observed. The average AIML length was comparable between male and female cadavers (Figure 1 and Figure 2). There was a slight trend toward male cadavers having a longer AIML, but this did not reach significance. For the right knees, male cadavers showed a mean length of about 34.9 mm compared to 34.2 mm in female cadavers (right knee: t(110)=1.64; p=0.10). For the left knees, male cadavers showed a mean length of 34.9 mm compared to 34.8 mm in female cadavers (left knee: t(110)=0.92; p=0.36) (Table 1). These findings suggest that AIML length appears consistent across sexes, reinforcing the notion that this ligament maintains a stable anatomical presence regardless of gender-related morphological differences.

**Table 1:** Measurements of average AIML and soft tissue architecture between male and female cadavers Each table includes the t-value and degrees of freedom (df=110) alongside p-values for all statistical tests. An unpaired t-test was used for all group comparisons, and statistical significance was set at p<0.05. Trends toward significance (p-values between 0.05 and 0.10) were noted, though not considered statistically significant. Comparison groups were defined as male vs. female measurements in both right and left knees. AIML: anterior intermeniscal ligament; ACL: anterior cruciate ligament; M: male; F: female; R: right; L: left

Average age (M=77; F=77.1)	Measurement	Male (R) (mean±SD)	Female (R) (mean±SD)	t-value (R)	p-value (R)	Male (L) (mean±SD)	Female (L) (mean±SD)	t-value (L)	p-value (L)
Length of AIML (mm)		34.87±6.03	34.18±4.34	1.637	0.10	34.90±4.87	34.78±4.89	0.9232	0.36
AIML thickness (mm)	Medial	1.46±0.49	1.19±0.37	2.1353	0.04	1.25±0.61	1.17±0.47	-1.1758	0.24
	Mid-point	1.26±0.54	1.11±0.45	1.6486	0.10	1.23±0.53	1.10±0.47	1.47953	0.14
	Lateral	1.24±0.46	1.25±0.30	0.5685	0.57	1.21±0.50	1.26±0.49	-0.263511	0.79
Anterior meniscal horn thickness (mm)	Medial horn	2.17±1.05	2.50±0.83	-1.80985	0.07	2.25±0.82	2.52±0.74	-1.1342	0.26
	Lateral horn	2.26±0.91	2.43±0.92	-0.249373	0.80	2.37±0.69	2.44±0.73	-0.5833	0.56
Posterior meniscal horn to AIML (mm)	Medial	28.57±6.43	31.11±6.55	-2.2885	0.20	31.69±5.15	31.69±7.79	-1.1168	0.27
	Lateral	26.27±7.13	29.08±5.83	-2.14156	0.30	30.22±4.14	30.32±7.57	0.26308	0.79
AIML to ACL (mm)		10.96±1.79	11.37±3.23	-0.64477	0.52	12.08±3.97	11.42±2.95	1.04115	0.30

AIML thickness

When comparing AIML thickness, notable trends emerged, particularly in the medial region. Male cadavers demonstrated significantly greater medial AIML thickness in the right knee compared to female cadavers, marking one of the few statistically significant differences detected in the study (t(110)=2.14; p=0.04) (Table 1). However, no significant difference was observed in the left knee (t(110)=-1.18; p=0.24). At the mid-point of the AIML, male cadavers tended to have slightly greater thickness than female cadavers, though this did not reach statistical significance (right knee: t(110)=1.65 and p=0.10; left knee: t(110)=1.48 and p=0.14). Similarly, lateral AIML thickness showed no significant differences between sexes in either knee (right knee: t(110)=0.57 and p=0.57; left knee: t(110)=-0.26 and p=0.79). The trend of male cadavers exhibiting greater medial AIML thickness suggests anatomical variation that may warrant further investigation. These differences could have clinical implications for joint mechanics, particularly in how the AIML distributes axial load during knee movement. 

Additional spatial relationships

Our remaining measurements assessed AIML's spatial relationships with adjacent structures, including the medial and lateral thickness of the anterior meniscal horn, the distance between the posterior meniscal horn and the AIML, and the distance from the AIML to the ACL root. While no statistically significant differences were observed, trends indicated that male cadavers had thinner medial and lateral meniscal horns, whereas female cadavers exhibited a greater distance between the AIML and the posterior meniscal horn. The measured distance from the AIML to the ACL root also showed no notable differences between groups (right knee: t(110)=-0.64 and p=0.52; left knee: t(110)=1.04 and p=0.30) (Table 1). These subtle anatomical variations may influence meniscal function and stability, highlighting the potential importance of sex-based differences in the structural organization of the knee joint.

## Discussion

The role of the AIML remains a topic of ongoing debate and is not fully understood. Our collected data was comparable to previously researched papers, specifically for the length of the AIML and the AIML to the base of the ACL [2,4,13]. Our study found no anatomical differences between male and female cadavers. The authors initially hypothesized that men would have a thicker AIML, similar to their lower rate of osteoarthritic prevalence in comparison to women [14]. Interestingly, no statistical difference was found, although men, on average, had thicker AIMLs overall, except in the lateral aspect of the ligament. While this trend did not reach statistical significance, it is possible that with a larger sample size of male specimens, a significant difference might have emerged. If truly different, these trends could be influenced by sex-related factors, such as the effects of estrogen on ligamentous degradation. Alternatively, it may suggest that the AIML plays a smaller role in reducing axial load than previously thought [9]. Future studies could investigate other sex-related factors, such as q-angles or estrogen exposure, and their effect on AIML morphology. Additionally, other avenues of research could investigate and compare morphological measurements in younger and older populations to evaluate whether this structure is sensitive to osteoarthritic changes, better assessing its functional role in knee joint stability.

A deeper understanding of the anatomical dimensions of the AIML and its relationship to surrounding structures could prove valuable during procedures, such as meniscal repair, meniscectomy, ACL reconstruction, and knee osteotomy, where its proximity should be carefully considered. A precise knowledge of these anatomical features allows for more accurate surgical planning, reducing the risk of inadvertent damage to the ligament and potentially preventing conditions like anterior pain syndrome [15]. This is further validified by previous research on the complex neural network of AIML likely playing a crucial role in knee joint integrity and function [10-12]. 

This study has several limitations that should be considered. Some cadaveric knees were previously dissected by medical students, rather than the researchers themselves, which may have introduced variability in tissue integrity and overall anatomical preservation. Additionally, cadavers with total knee replacements were excluded from the study, limiting the generalizability of findings to patients with intact native knee structures. Lastly, the relatively low sample size of this study may affect the statistical power of the results. A larger sample size could help to enhance the study's reliability and allow for a more comprehensive understanding of the anatomical and clinical significance of the AIML.

## Conclusions

Our study contributes valuable information to the existing body of knowledge on the AIML, particularly in terms of precise anatomical measurements and spatial relationships. We found that the AIML is present and of similar length between male and female specimens. The ligament's thickness is also comparable between sexes, aside from a modest increase in medial thickness observed in male knees. We quantified the distances from the AIML to key structures like the ACL and meniscal horns, finding no significant sex differences in these spatial relationships. 

Clinicians and anatomists can use this information to enhance knee examinations, and surgeons can use it to avoid iatrogenic injury to the AIML. Preserving critical structures like the AIML during knee surgery may help maintain normal joint biomechanics and potentially improve post-operative outcomes. With a comprehensive appreciation of AIML's anatomy and function, we can better protect the integrity of the knee joint and support its complex load-bearing and sensory roles. 
